# Spatial variability of the Po River food web and its comparison with the Danube River food web

**DOI:** 10.1371/journal.pone.0288652

**Published:** 2023-07-14

**Authors:** Katalin Patonai, Ferenc Jordán, Giuseppe Castaldelli, Leonardo Congiu, Anna Gavioli

**Affiliations:** 1 Department of Environmental and Prevention Sciences, University of Ferrara, Ferrara, Italy; 2 Department of Plant Systematics, Ecology and Theoretical Biology, Eötvös Loránd University, Budapest, Hungary; 3 Department of Chemistry, Life Sciences and Environmental Sustainability, University of Parma, Parma, Italy; 4 Department of Biology, University of Padua, Padova, Italy; Institute for Biological Research, University of Belgrade, SERBIA

## Abstract

Freshwater ecosystems are experiencing unprecedented pressure globally. To address environmental challenges, systematic and comparative studies on ecosystems are needed, though mostly lacking, especially for rivers. Here, we describe the food web of the Po River (as integrated from the white literature and monitoring data), describe the three river sections using network analysis, and compare our results with the previously compiled Danube River food web. The Po River food web was taxonomically aggregated in five consecutive steps (T1-T5) and it was also analyzed using the regular equivalence (REGE) algorithm to identify structurally similar nodes in the most aggregated T5 model. In total, the two river food webs shared 30 nodes. Two network metrics (normalized degree centrality [nDC]) and normalized betweenness centrality [nBC]) were compared using Mann-Whitney tests in the two rivers. On average, the Po River nodes have larger nDC values than in the Danube, meaning that neighboring connections are better mapped. Regarding nBC, there were no significant differences between the two rivers. Finally, based on both centrality indices, *Carassius auratus* is the most important node in the Po River food web, whereas phytoplankton and detritus are most important in the Danube River. Using network analysis and comparative methods, it is possible to draw attention to important trophic groups and knowledge gaps, which can guide future research. These simple models for the Po River food web can pave the way for more advanced models, supporting quantitative and predictive—as well as more functional—descriptions of ecosystems.

## Introduction

Global environmental challenges call for systematic and comparative studies on ecosystems. This requires standard sampling methods, data management, and well-established methods providing results that are either testable or (at least) provide support for planning empirical work. Despite of large number of field campaigns and huge amount of primary data, it is still quite in infancy to perform fully comparable analyses on several ecosystems, even if of similar nature. This needs integration of knowledge from environmental and social sciences, statistics, computational ecology, and data science (e.g., coordinated sampling and data sharing protocols [1, 2]).

Overall, the biodiversity of freshwater ecosystems is experiencing unprecedented pressure globally, including climate change and multiple threats combined, such as invasive species, altered flow regulation, land-use change, pollution, and overexploitation [3, 4]. These combined effects put freshwater ecosystems under increased vulnerability, while at the same time, humans also rely on these ecosystems as a water source, major transportation routes, and for recreational activities [5]. Whereas some freshwater ecosystems (mostly lakes) have been extensively monitored over the years (e.g., Lake Constance [6, 7]), rivers in general are underrepresented in ecosystem modelling (for instance only a handful of Ecopath with Ecosim [EwE] river models are available [8]). In this study, we addressed this problem by describing the food web of the Po River ecosystem, following the methodology of an earlier study on the Danube River [9]. We focus on the availability of published data (i.e., white literature), the challenges of data aggregation and food web construction, as well as comparative analysis of the two river ecosystems. Macroscopic indicators at the ecosystem level (e.g., network analysis) should be incorporated in standard monitoring protocols [10], given that various organisms give contrasting responses (i.e., of mixed sign) to selective effects.

Given the regional importance of the Po River that is the largest basin in Italy, it becomes necessary to examine the river ecosystem at the network-level. A system-level approach allows the integration of the network parts (species or major functional groups) and their connections (e.g., predator-prey interactions) [11, 12]. By looking at the complex ecosystem (multi-species, rather than single species, approach), it is possible to find emergent ecosystem properties [13, 14], ecological indicators [4, 15] or keystone species [5, 16]. These increase our understanding of the functioning of an ecosystem, identify important species, or highlight problems, such as knowledge gaps. Network-based approach can thus be used to summarize and intergrate available data that then provide useful insights.

There are several types of ecosystem models [17, 18]. In this study, we focus on trophic networks (food webs) in which consumers are linked with resources [19]. We aimed to establish simple connectance webs (based on presence/absence of predator-prey links) of the Po River ecosystem and its three main river sections. An initial step here is establishing the known nodes and their connections [20] which was done using a literature search (Web Of Science), a book on Italian fish [21], and field monitoring survey data. Our aims were to i) describe each section of the river by mapping the connectance webs; ii) aggregate the raw (heterogenous) networks into more compact models, based on taxonomic aggregation following previously described methods [9, 22]; iii) describe the river sections using network analysis; and iv) compare the Po River food web with the Danube River food web, the second largest river in Europe [9]. In food webs, it is also possible to aggregate nodes based on structural similarity, which create”trophic guilds” [23–25], as an aggregation from an ecological perspective. The structure of the final aggregated network was also analysed using the regular equivalence (REGE) algorithm [24]. These first steps will pave the way for more advanced models [see 26, 27], supporting quantitative and predictive as well as more functional descriptions of ecosystems [28].

## Methods

### The Po River ecosystem

**Fig 1:**
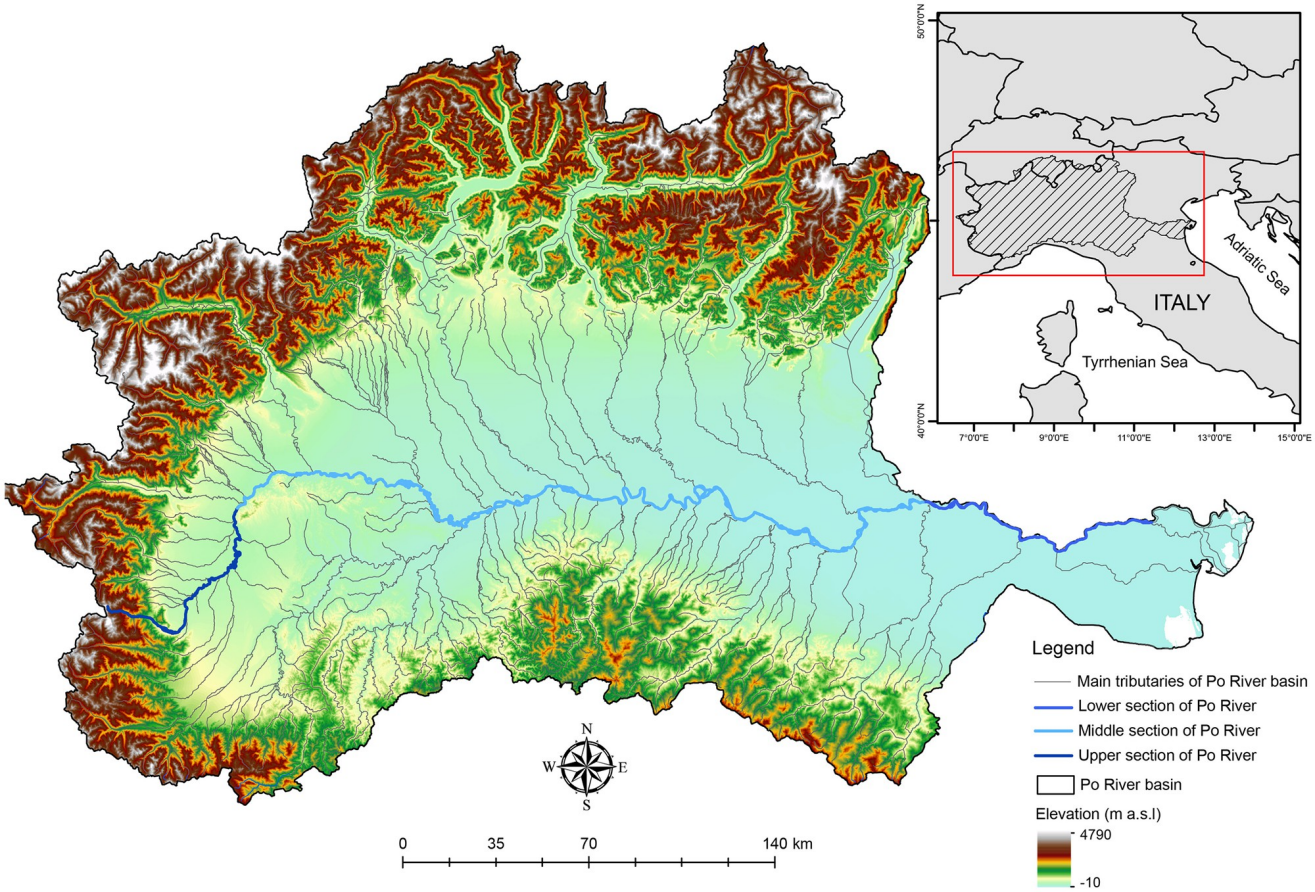
Map of the Po River basin with the three river sections: The Upper section of the Po River (in blue), the Middle section of the Po River (in cyan) and the Lower Po River (in light blue). The main tributaries of Po River (in grey) and the elevation of the basin are also shown (based on open source shapefiles downloaded from European Environment Agency, https://www.eea.europa.eu/en/analysis).

Located in the Mediterranean region, the Po River is a major river with a length of 652 km and with a drainage area of ~71000 km^2^, between the Alps and the Italian Apennines (Fig 1). Its area is almost one-fourth that of Italy and where 40% of the Italian GDP is produced [29]. Based on environmental homogeneity and aquatic community composition, we identified three sections in the Po River (Fig 1). The Upper Po River section from the source to Turin city (125 km long) is characterized by cold and turbulent waters, limited water flow, steep slope, predominantly boulder and pebble bed until the slope weakens and with it the turbulence and coarseness of the substrate, allowing colonization by rheophilic cyprinids. The Middle Po River section from Turin city to the Secchia River confluence (543 km long) is characterized by low slope, higher flow rate, general heating of the water, the connection to a system of lateral (off-channel) habitats, that are beginning to become complex and they assume an important role for the river ecosystem itself, and which in turn enhances species richness. Finally, the Lower Po River section, from the Secchia River confluence to the Po River Delta (127 km long), is characterized by deep, warmer and slower flow and less surface turbulence waters compared to other river sections, with the presence of euryhaline species. The high ecological, as well as socio-economic, value make the Po River a case study of European importance.

### Data

Initially, a Web Of Science literature survey was conducted using the "Po River" term and the following keywords: "food web", "interaction", "prey", "predator, "network", "trophic", "feeding", "gut content", "isotope", "diet" (accesssed on April 28, 2022). This search found 279 articles. Studies on terrestrial species and interactions (e.g., Po floodplain) were not considered. The abstracts were screened, from which only 7 articles were found to be relevant. After reviewing these selected papers, two articles had data on rotifer predator-prey interactions [30, 31]; two papers had detailed species list, but lacking diet information [32, 33]; and three papers had information on plankton and macroinverterbrate community composition [34–36]. Therefore, it was not possible to obtain data for the Po River food web based only on white literature. Our research was complemented with survey data for fish, invertebrates, and plankton (see S1 Table for references). Information on predator-prey relationships was obtained from FishBase [37] and from published books [21, 38]. Where available, size information (small/large) and age information (juvenile/adult) were added to the database.

### Network construction and analyses

Data was compiled for each river section (i.e., Upper, Middle, and Lower Po River sections) separately and merged into a master network (S2 Table). The network nodes (species or functional groups) are listed exactly as mentioned in the original sources. We decided to exclude the two articles specifically focused on the rotifera community [30, 31], because they would have produced an isolated rotifera network (rotifers eating other rotifers). Although this subsystem is indeed very important, it realistically needs to be linked to the rest of the community, thus is something that needs further future consideration.

After compiling all data, we followed the taxonomic aggregation procedure detailed in Patonai and Jordán (2021) for the Danube River [9], for comparability and standardization of methods. This includes five consecutive steps (Table 1) with a minor modification at step two (having detailed fish data, fish species were also aggregated into taxonomic families). In the first step (T1), the master dataset was aggregated so that the size (small/large) and age (juvenile/adult) information was combined at species level. In the second step (T2), we combined fish species into fish families (e.g., *Silurus glanis* into Siluridae). In the third step (T3), the two main fish orders were created (Cypriniformes, Perciformes). In step four (T4), invertebrates were aggregated into main groups (Annelida, Gastropoda, Mollusca, Crustacea, Insecta, Turbellaria). Finally, in step five (T5), all producers were aggregated, including nodes such as algae, diatoms, phytoplankton, and macrophytes. The detritus node remained separate (unaggregated). We note that the aggregation steps can be done in any order (since they represent separate taxonomic groups) and the effect of each aggregation step can be quantified using network analysis. We followed this order, because it logically goes from the smallest (species-level aggregation), through intermediate steps (taxonomic family, order, class), to the broadest category (producers).

**Table 1:** List of node codes and node names. Presence is marked with an ‘x’. The Master list contains all nodes as they appeared in the original sources. The aggregation steps (T1-T5) show nodes belonging to the five taxonomical aggregation steps.

code	node name	Master	T1	T2	T3	T4	T5
1	*Abramis brama*	x	x	x			
2	*Acanthocyclops gr*. *vernalis-robustus*	x	x	x	x		
3	*Acanthocyclops spp*	x	x	x	x		
4	*Acipenser naccarii*	x	x				
5	*Alburnus arborella*	x	x	x			
6	ALGAE	x	x	x	x	x	
7	*Alona guttata*	x	x	x	x		
8	AMPHINEMURA	x	x	x	x		
9	ANCYLIDAE	x	x	x	x		
10	*Anguilla anguilla*	x	x				
11	*Anguilla anguilla* LARGE	x					
12	*Anguilla anguilla* SMALL	x					
13	APHELOCHEIRIDAE	x	x	x	x		
14	ASELLIDAE	x	x	x	x		
15	ASTACIDAE	x	x	x	x		
16	ATHERICIDAE	x	x	x	x		
17	BAETIDAE	x	x	x	x		
18	BAETIS	x	x	x	x		
19	*Barbus barbus*	x	x	x			
20	*Barbus plebejus*	x	x	x			
21	*Barbus tyberinus*	x	x	x			
22	BATRACOBDELLA	x	x	x	x		
23	BERAEIDAE	x	x	x	x		
24	BLEPHARICERIDAE	x	x	x	x		
25	*Blicca bjoerkna*	x	x	x			
26	*Bosmina longirostris*	x	x	x	x		
27	BRACHYPTERA	x	x	x	x		
28	BYTHINIIDAE	x	x	x	x		
29	*Bythotrephes*	x	x	x	x		
30	CAENIDAE	x	x	x	x		
31	CAENIS	x	x	x	x		
32	CALOPTERYX	x	x	x	x		
33	CAPNIA	x	x	x	x		
34	*Carassius auratus*	x	x	x			
35	CENTROPTILUM	x	x	x	x		
36	CERATOPOGONIDAE	x	x	x	x		
37	*Ceriodaphnia pulchella*	x	x	x	x		
38	CHIRONOMIDAE	x	x	x	x		
39	CHLOROPERLA	x	x	x	x		
40	*Chondrostoma soetta*	x	x	x			
41	CHOROTERPES	x	x	x	x		
42	*Chydorus ovalis*	x	x	x	x		
43	*Chydorus sphaericus*	x	x	x	x		
44	CLADOCERA	x	x	x	x		
45	CLOEON	x	x	x	x		
46	*Cobitis bilineata*	x	x	x			
47	COENAGRION	x	x	x	x		
48	CORIXIDAE	x	x	x	x		
49	*Cottus gobio*	x	x				
50	CRANGONYCTIDAE	x	x	x	x		
51	CRENOBIA	x	x	x	x		
52	CULICIDAE	x	x	x	x		
53	*Cyclops vicinus*	x	x	x	x		
54	*Cyprinus carpio*	x	x	x			
55	CYSTOBRANCHUS	x	x	x	x		
56	DAPHNIA	x	x	x	x		
57	*Daphnia ambigua*	x	x	x	x		
58	*Daphnia galeata*	x	x	x	x		
59	*Daphnia gr*. *Longispina*	x	x	x	x		
60	*Daphnia hyalina*	x	x	x	x		
61	DENDROCOELUM	x	x	x	x		
62	Detritus	x	x	x	x	x	
63	*Diacyclops*	x	x	x	x		
64	*Diaphanosoma*	x	x	x	x		
65	*Diaphanosoma brachyurum*	x	x	x	x		
66	*Diaptomus*	x	x	x	x		
67	DIATOMS	x	x	x	x		
68	DINA	x	x	x	x		
69	DINOCRAS	x	x	x	x		
70	DIPTERA	x	x	x	x		
71	DIXIDAE	x	x	x	x		
72	DRYOPIDAE	x	x	x	x		
73	DUGESIA	x	x	x	x		
74	DYTISCIDAE	x	x	x	x		
75	ECDYONURUS	x	x	x	x		
76	ELECTROGENA	x	x	x	x		
77	ELMIDAE	x	x	x	x		
78	EMPIDIDAE	x	x	x	x		
79	ENCHYTRAEIDAE	x	x	x	x		
80	EPEORUS	x	x	x	x		
81	EPHEMERA	x	x	x	x		
82	EPHEMERELLA	x	x	x	x		
83	EPHORON	x	x	x	x		
84	EPHYDRIDAE	x	x	x	x		
85	Ergasilidae	x	x	x	x		
86	ERPOBDELLA	x	x	x	x		
87	*Esox cisalpinus*_ADULTS	x					
88	EUBRIIDAE	x	x	x	x		
89	*Eudiaptomus gracilis*	x	x	x	x		
90	*Eudiaptomus padanus*	x	x	x	x		
91	*Gambusia holbrooki*	x	x				
92	GAMMARIDAE	x	x	x	x		
93	GLOSSIPHONIA	x	x	x	x		
94	GLOSSOSOMATIDAE	x	x	x	x		
95	*Gobio gobio*	x	x	x			
96	GOERIDAE	x	x	x	x		
97	GORDIIDAE	x	x	x	x		
98	*Gymnocephalus cernua*	x	x	x			
99	GYRINIDAE	x	x	x	x		
100	HABROLEPTOIDES	x	x	x	x		
101	HABROPHLEBIA	x	x	x	x		
102	HALIPLIDAE	x	x	x	x		
103	HAPLOTAXIDAE	x	x	x	x		
104	HELICOPSYCHIDAE	x	x	x	x		
105	HELOBDELLA	x	x	x	x		
106	HELODIDAE	x	x	x	x		
107	HELOPHORIDAE	x	x	x	x		
108	HEMICLEPSIS	x	x	x	x		
109	HEPTAGENIA	x	x	x	x		
110	HIRUDO	x	x	x	x		
111	HYDRACARINA	x	x	x	x		
112	HYDRAENIDAE	x	x	x	x		
113	HYDROBIOIDAEA	x	x	x	x		
114	HYDROPHILIDAE	x	x	x	x		
115	HYDROPSYCHIDAE	x	x	x	x		
116	HYDROPTILIDAE	x	x	x	x		
117	HYGROBIIDAE	x	x	x	x		
118	ISOPERLA	x	x	x	x		
119	LEPIDOSTOMATIDAE	x	x	x	x		
120	LEPIDOPTERA	x	x	x	x		
121	*Lepomis gibbosus*	x	x	x			
122	LEPTOCERIDAE	x	x	x	x		
123	LESTES	x	x	x	x		
124	*Lethenteron zanandreai*	x	x				
125	*Leuciscus aspius*	x	x	x			
126	*Leucos aula*	x	x	x			
127	LEUCTRA	x	x	x	x		
128	LIBELLULA	x	x	x	x		
129	LIMNEPHILIDAE	x	x	x	x		
130	LIMONIIDAE	x	x	x	x		
131	*Chelon ramada (formerly Liza ramada)*	x	x				
132	LUMBRICIDAE	x	x	x	x		
133	LUMBRICULIDAE	x	x	x	x		
134	LUMNAEIDAE	x	x	x	x		
135	MACROPHYTES	x	x	x	x	x	
136	*Macrothrix laticornis*	x	x	x			
137	*Mesocyclops leuckarti*	x	x	x			
138	MESOVELIIDAE	x	x	x	x		
139	*Micropterus salmoides*	x	x	x			
140	*Misgurnus anguillicaudatus*	x	x	x			
141	*Moina micrura*	x	x	x	x		
142	NAIDIDAE	x	x	x	x		
143	NAUCORIDAE	x	x	x	x		
144	NEMOURA	x	x	x	x		
145	NEPIDAE	x	x	x	x		
146	NERITIDAE	x	x	x	x		
147	NIPHARGIDAE	x	x	x	x		
148	NOTONECTIDAE	x	x	x	x		
149	OCHTERIDAE	x	x	x	x		
150	ODONTOCERIDAE	x	x	x	x		
151	OLIGONEURIELLA	x	x	x	x		
152	ONYCHOGOMPHUS	x	x	x	x		
153	OSTRACODA	x	x	x	x		
154	*Padogobius bonelli*	x	x	x			
155	PARALEPTOPHLEBIA	x	x	x	x		
156	*Perca fluviatilis*_ADULTS	x					
157	*Perca fluviatilis*_JUVENILES	x					
158	PERLA	x	x	x	x		
159	PERLODES	x	x	x	x		
160	PHILOPOTAMIDAE	x	x	x	x		
161	*Phoxinus phoxinus*	x	x	x			
162	PHYRRHOSOMA	x	x	x	x		
163	PHYSIDAE	x	x	x	x		
164	Phytoplankton	x	x	x	x	x	
165	PISCICOLA	x	x	x	x		
166	PISCICOLIDAE	x	x	x	x		
167	PISIDIIDAE	x	x	x	x		
168	PLANORBIDAE	x	x	x	x		
169	PLEIDAE	x	x	x	x		
170	*Pleuroxus aduncus*	x	x	x	x		
171	*Pleuroxus denticulatus*	x	x	x	x		
172	POLYCELIS	x	x	x	x		
173	POLYCENTROPODIDAE	x	x	x	x		
174	POTAMANTHUS	x	x	x	x		
175	POTAMIDAE	x	x	x	x		
176	PROCLOEON	x	x	x	x		
177	PROPAPPIDAE	x	x	x	x		
178	*Protochondrostoma genei*	x	x	x			
179	PROTONEMURA	x	x	x	x		
180	*Pseudorasbora parva*	x	x	x			
181	PSYCHODIDAE	x	x	x	x		
182	PSYCHOMYIIDAE	x	x	x	x		
183	PYRGULIDAE	x	x	x	x		
184	RHABDIOPTERYX	x	x	x	x		
185	RHITHROGENA	x	x	x	x		
186	*Rhodeus sericeus*	x	x	x	x		
187	RHYACOPHILIDAE	x	x	x	x		
188	*Rutilus rubilio*	x	x	x			
189	*Rutilus rutilus*	x	x	x			
190	*Salaria fluviatilis*	x	x	x			
191	*Salmo marmoratus*_ADULTS	x					
192	*Salmo marmoratus*_JUVENILES	x					
193	*Salmo trutta*_SMALL	x					
194	*Salmo trutta*_LARGE	x					
195	*Sander lucioperca*	x	x	x			
196	*Scapholeberis mucronata*	x	x	x	x		
197	*Scardinius erythrophthalmus*	x	x	x			
198	SERICOSTOMATIDAE	x	x	x	x		
199	*Silurus glanis*_LARGE	x					
200	*Silurus glanis*_SMALL	x					
201	*Simocephalus vetulus*	x	x	x	x		
202	SIMULIIDAE	x	x	x	x		
203	SIPHONOPERLA	x	x	x	x		
204	SPHAERIIDAE	x	x	x	x		
205	*Squalius cephalus*	x	x	x			
206	*Squalius squalus*	x	x	x			
207	STRATIOMYIDAE	x	x	x	x		
208	SYMPECMA	x	x	x	x		
209	TABANIDAE	x	x	x	x		
210	TARNETRUM	x	x	x	x		
211	*Telestes muticellus*	x	x	x			
212	*Thermocyclops crassus*	x	x	x	x		
213	*Thymallus thymallus*	x	x				
214	*Tinca tinca*	x	x	x			
215	TIPULIDAE	x	x	x	x		
216	TUBIFICIDAE	x	x	x	x		
217	UNIONIDAE	x	x	x	x		
218	VALVATIDAE	x	x	x	x		
219	VIVIPARIDAE	x	x	x	x		
220	*Esox cisalpinus*		x				
221	*Perca fluviatilis*		x	x			
222	*Salmo marmoratus*		x				
223	*Salmo trutta*		x				
224	*Silurus glanis*		x				
225	Cypriniformes				x	x	x
226	Perciformes				x	x	x
227	Acipenseridae			x	x	x	x
228	Anguillidae			x	x	x	x
229	Cottidae			x	x	x	x
230	Cyprinodontidae			x	x	x	x
231	Esocidae			x	x	x	x
232	Mugilidae			x	x	x	x
233	Petromyzontidae			x	x	x	x
234	Salmonidae			x	x	x	x
235	Siluridae			x	x	x	x
236	Annelida					x	x
237	Copepoda					x	x
238	Amphipoda					x	x
239	Crustacea					x	x
240	Gastropoda					x	x
241	Mollusca					x	x
242	Insecta					x	x
243	Turbellaria					x	x
244	Producers						x

Prior to analyses, binary (unweighted) networks were made symmetric (taking the interaction sums between two nodes, x_*ij*_ + x_*ji*_) in UCINET software [39]. Network visualization was done in R software version 4.2.0 [40] using Sankey plots in the ‘networkD3’ package [41]. The final aggregated (T5) river section networks were also clustered using the regular equivalence (REGE) algorithm, which groups nodes based on topological similarity [24, 42], essentially quantifying trophic guilds based on food web structure [23].

#### Global network metrics

Global network metrics were computed in UCINET software [39]. Six global metrics were computed: number of nodes (N), number of links (L), network density (D), clustering coefficient (CL), average path length (d), and small-world index (SW). Number of nodes (N) is the number of species or functional groups in the network. Number of links (L) describes the number of connections between the nodes. Both of these measures give an indication of the complexity of the network. Network density (D) or connectance is the number of actual links divided by the number of possible links [43]. It gives a quick snapshot of specialists vs generalists in the network (e.g., more generalists increase density, but more specialists decrease density). Network density is computed as D = 2L/N(N-1), where N (number of nodes) and L (number of links). Clustering coefficient (CL) is a measure of cohesion, it is the probability that two neighboring nodes *n* and *m* also share the neighbor node *i*. The weighted overall clustering coefficient reported is the”weighted mean of the clustering coefficient of all the nodes each one weighted by its degree” [39]. The average path length (d) is the mean distance between each node. In unweighted connectance webs, the distance is simply the number of links between two nodes. With shorter distance values, network effects can spread faster. Finally, the small-world index (SW) is computed by dividing CL by d. Small SW values characterize random graphs (small CL and d values), intermediate SW values characterize small-world networks (e.g., high CL and small d), and the higher SW values are typical for regular graphs (e.g., a lattice having large CL and d values). Networks found in nature are found between the spectrum of random and regular graphs [44].

#### Local network metrics

Two local network centrality measures were computed. Normalized degree centrality (nDC) describes each node by the number of direct connections of a given node divided by N-1 [43]. It indicates how richly connected is a particular node considering its immediate neighborhood. Normalized betweenness centrality (nBC) for node *i* is the number of shortest paths between each pair of node *j* and *k*, containing node *i* [43]. It indicates a particular node’s importance, in which large values suggest that the node acts as a bridge within the network structure. These two metrics were calculated in UCINET [39].

### Comparison with the Danube River trophic network

The Danube is the second longest river in Europe, traversing over several countries from the Black Forest in Germany to the Black Sea in Romania [45]. Similarly to the Po River, it also has three main river sections [45] and a connectance web has been already compiled [9], which makes it possible to compare the two rivers. We were interested in what information is available for both rivers and what are the knowledge gaps. The two rivers were compared using the previously described local network metrics (nDC, nBC). Comparative and standardized methodology is crucial when comparing ecosystems.

## Results and discussion

**Fig 2:**
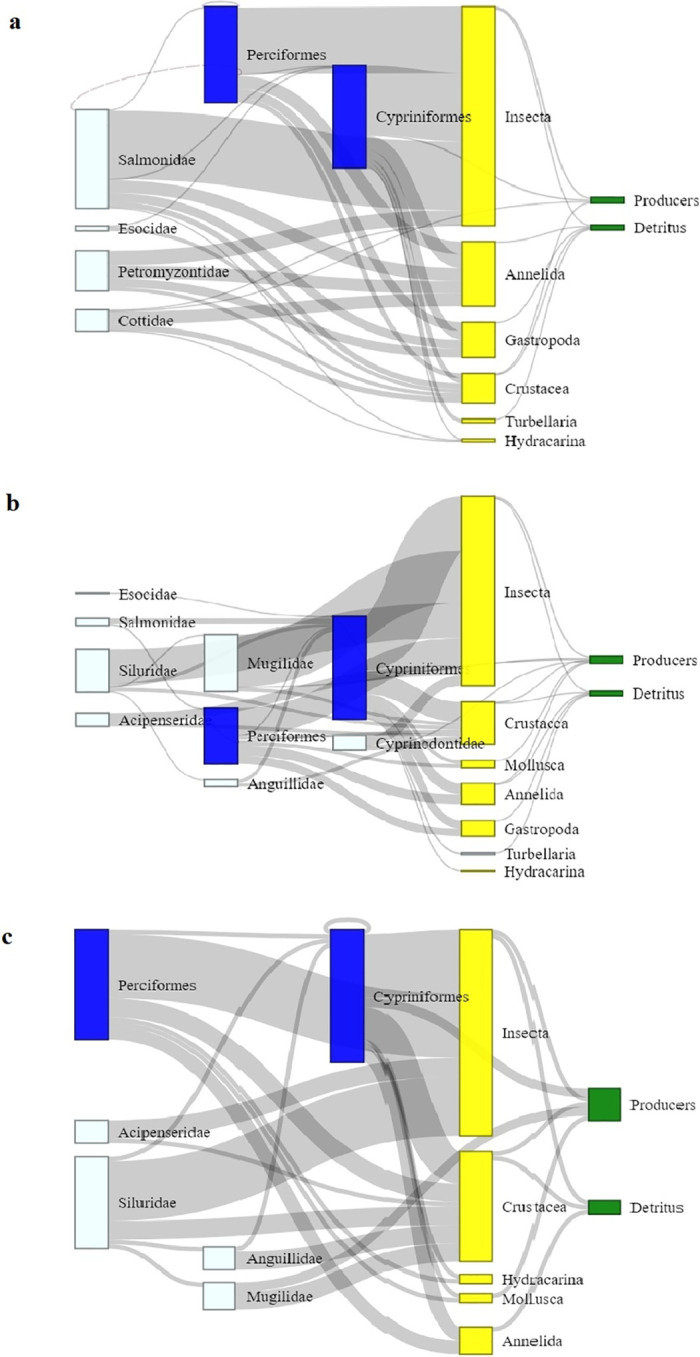
Sankey plots showing the food webs for the (a) Upper, (b) Middle, and (c) Lower sections of the Po River, composed of functional groups resulting from the aggregation process. In each color, the T2-T5 aggregation steps are indicated (T1: size and age aggregation, not relevant here; T2: Perciformes, Cypriniformes; T3: fish families; T4: invertebrates; T5: producers). The width of the flows (grey connectors) indicates the number of nodes (species or functional groups) that have been aggregated from the original networks. The self-loop in the Lower Po indicates cannibalism (Cypriniformes eating other Cypriniformes).

The global network metrics and the aggregation steps are summarized in Table 2 and visualized in Sankey plots (Fig 2). The global metrics give a snapshot of the consecutive networks. The Middle Po River had the most nodes and links, the Upper Po was comparable, and the Lower Po River had the fewest nodes and links (Table 2). Small fluctuations in the first steps were insignificant, whereas the changes in network metrics in the final aggregation steps (T4-T5) were more substantial. The T4 and T5 aggregation steps involved taxonomically diverse groups (invertebrates, producers), hence they had large effect on the networks. In each case, the number of nodes (N) and links (L) monotonically decreased through the aggregation process. The aggregation made the network nodes more homogenous. Network density (D) and clustering coefficient (CL) were generally variable in the first steps, but then increased in the last steps (T3-T5), making the final networks more dense and clustered. The average distance (d) decreased in all cases, except in the Upper Po, where it slightly increased at T2 and T3, but then decreased. Small-world index (SW) increased during the aggregation steps, ranging from *i*) very small values descriptive of random networks; to *ii*) larger numbers in the more aggregated versions, descriptive of other natural networks [44]. These global metrics are comparable in all three river sections and the master network, indicating that the most aggregated networks can be described as clustered (high density and clustering coefficients with reduced average network distances), and they are the most comparable to natural networks (i.e., intermediate SW values).

**Table 2:** Global network metrics for the three river sections (Upper, Middle, Lower Po) and the combined Master food web, in each consecutive taxonomic aggregation step (T1-T5). Six global metrics were computed: N (number of nodes), L (number of links), D (network density), CL (weighted overall clustering coefficient), d (average distance), SW (small-world index).

	**Upper**	**T1**	**T2**	**T3**	**T4**	**T5**
N	149	146	144	127	16	14
L	1162	1162	1008	410	41	39
D	0.105	0.110	0.098	0.051	0.333	0.418
CL	0.002	0.053	0.037	0.056	0.271	0.323
d	2.367	2.311	2.325	2.349	1.783	1.648
SW	0.001	0.023	0.016	0.024	0.152	0.196
	**Middle**	**T1**	**T2**	**T3**	**T4**	**T5**
N	156	152	151	124	20	18
L	1274	1274	1266	377	46	44
D	0.105	0.111	0.112	0.049	0.232	0.275
CL	0.000	0.015	0.015	0.036	0.191	0.286
d	2.381	2.301	2.300	2.000	1.921	1.850
SW	0.000	0.007	0.007	0.018	0.099	0.155
	**Lower**	**T1**	**T2**	**T3**	**T4**	**T5**
N	58	55	55	37	16	13
L	314	314	314	108	32	30
D	0.190	0.211	0.211	0.161	0.258	0.372
CL	0.004	0.043	0.043	0.130	0.241	0.344
d	2.267	2.137	2.137	2.006	2.008	1.718
SW	0.002	0.020	0.020	0.065	0.120	0.200
	**Master**	**T1**	**T2**	**T3**	**T4**	**T5**
N	219	213	211	181	23	20
L	2229	2229	2074	681	65	61
D	0.093	0.099	0.094	0.042	0.249	0.311
CL	0.002	0.041	0.029	0.042	0.243	0.308
d	2.417	2.333	2.339	2.101	1.929	1.779
SW	0.001	0.018	0.012	0.020	0.126	0.173

**Fig 3:**
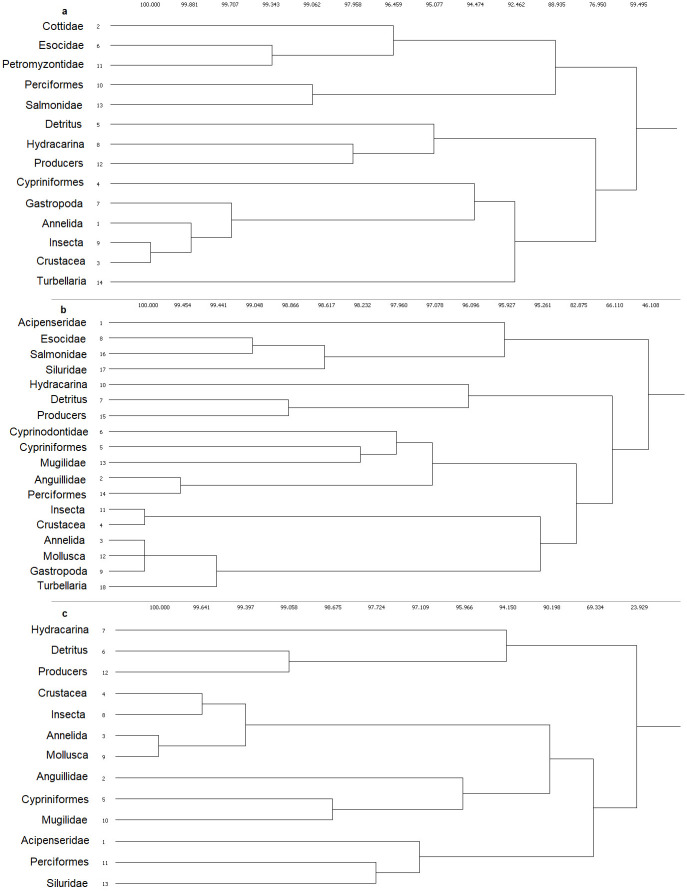
The REGE similarity dendrograms for the (a) Upper, (b) Middle, and (c) Lower sections, based on the final food webs (aggregated T5). Nodes are listed on the left, and the horizontal numbers indicate the similarity based on regular equivalence (100 = strict regular equivalence). Higher similarity (i.e., shorter distance in the dendrogram) between nodes *i* and *j* means that these nodes are structurally more similar (e.g., share similar direct and indirect neighbourhoods).

The regular equivalence (REGE) algorithm examined these networks from a different perspective. Results of the REGE clustering for each river section are found in Fig 3. The REGE algorithm highlights the level or structural similarity the nodes (organisms) have in each river section. For example, Insecta and Crustacea nodes have the same functional role in the three river sections. Hydracarina (water mites commonly found in freshwaters) are structurally grouped with detritus and producers (because all three nodes only have consumers eating them). This nicely shows that REGE highlights topological resemblance instead of the taxonomic approach. For the fish groups, REGE gave interesting insights. For some fish groups, the topological similarity reflected some difference from the taxonomically-derived Sankey plots. In the Upper Po River, Cypriniformes has closer resemblance to the invertebrate groups (probably due to being a predator as well as a prey), whereas the other five fish groups (i.e. Cottidae, Esocidae, Petromyzontidae, Perciformes, and Salmonidae) are grouped together into one cluster (Figs 2a and 3a). In the Middle Po River, the two methods were similar for fish (Figs 2b and 3b) resulting in two fish clusters (higher trophic level fish: Esocidae, Salmonidae, Siluridae, Acipenseridae; and intermediate- and lower trophic-level fish: Anguillidae, Mugilidae, Perciformes, Cypriniformes, and Cyprinodontidae). In the Lower Po River, the two main REGE fish clusters (higher trophic level fish, mostly predators: Perciformes, Acipenseridae, Siluridae; and intermediate trophic-level fish: Anguillidae, Mugilidae, Cypriniformes, Fig 3c) nicely match the Sankey plot (Fig 2c). The self-loop in the Lower Po indicates cannibalism (Cypriniformes eating other Cypriniformes) (Fig 2c). Overall, the REGE clusters provide additional structural support for the Sankey plots, which are purely used for visualization purposes. However, the Sankey plots are informative due to the width of the fluxes (representing the number of nodes associated with each aggregated group). In the Upper Po River, the main diet of the larger aggregate fish groups (e.g., Salmonidae, Perciformes, Cypriniformes) are insects more than other invertebrates (e.g., Crustacea, Gastropoda, Annelida) (Fig 2a). In the Middle Po River, this is still true, but the importance of Crustacea in the diet increased for Cypriniformes, though not for Perciformes (Fig 2b). In the Lower Po River, the overall importance of Crustacea in the diet of various fish groups increased (Fig 2c).

**Fig 4:**
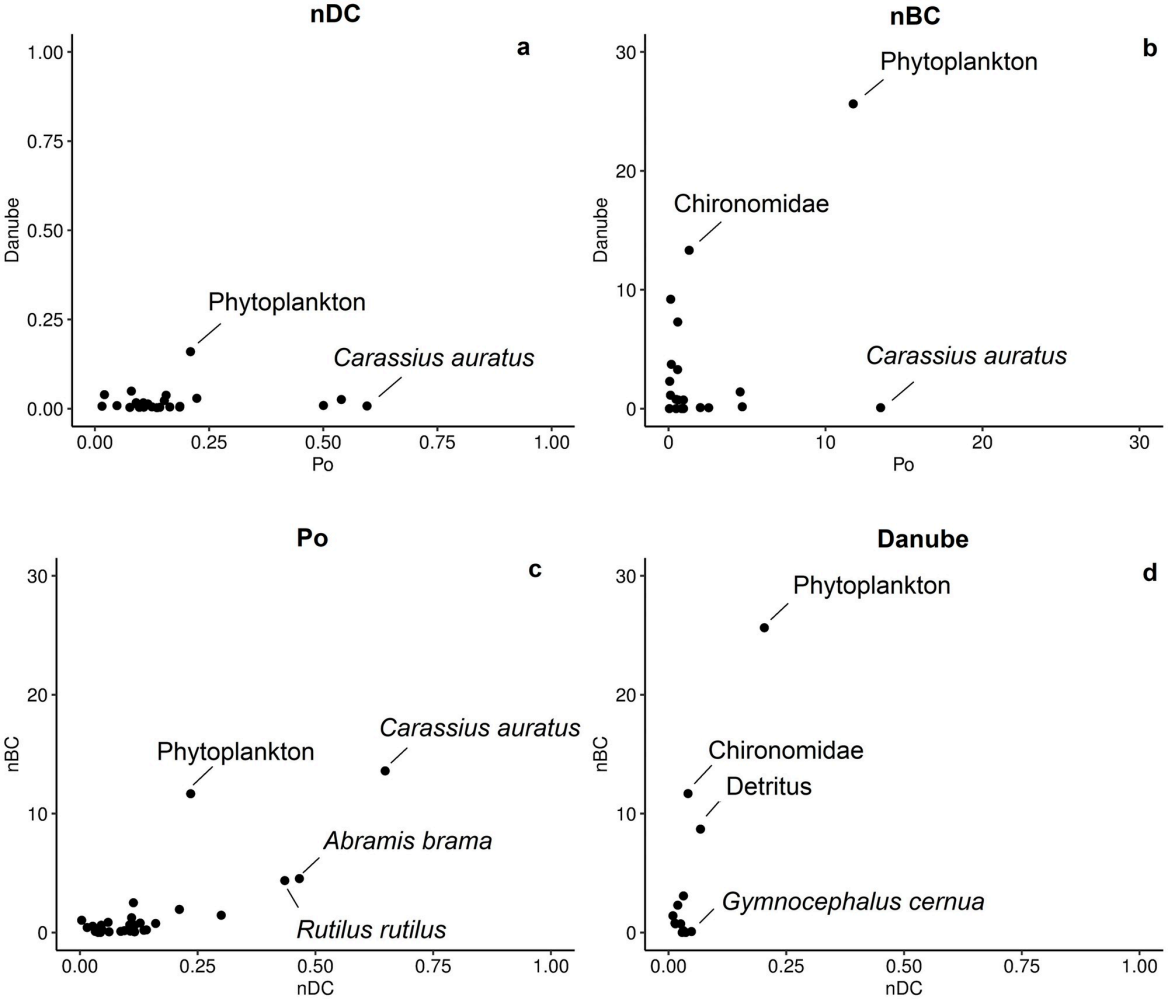
Local network metrics (nDC = normalized degree centrality, nBC = normalized betweenness centrality) for the Po River and the Danube River master network’s shared nodes (species or functional groups). (a) Normalized degree centrality (nDC) in both rivers, (b) normalized betweenness centrality (nBC) in both rivers, (c) nDC and nBC in the Po River, and (d) nDC and nBC in the Danube River. Correlation between these two indices is high (r_po_ = 0,76, r_danube_ = 0,93), reflecting also their mathematical relatedness. Points that are highly correlated indicate nodes that are important for both degree and betweenness centrality (e.g., *C*. *auratus* in the Po River, phytoplankton in the Danube), whereas others are more important for either nBC (e.g., Chironomidae in the Danube) or nDC (e.g., *G*. *cernua* in the Danube).

In comparing the Po River with the Danube River, we found that they share 30 nodes (Table 3). On average, the Po River nodes have larger nDC values than in the Danube (median_po_ = 0.110, median_danube_ = 0.008, *n* = 30, Mann-Whitney U = 52.5, *p*<0.0001), meaning that neighboring connections are better mapped in the Po River. Interestingly, the Danube River also had lower nDC values when comparing invasive species in Lake Balaton (Hungary) [9], suggesting that the Danube River is the least well mapped (e.g., least amount of data) or that these species are ecologically not as well connected in the Danube River. Some nodes have comparable nDC values in both rivers (e.g., phytoplankton, *Moina micrura*, *Sander lucioperca*, Table 3, Fig 4a), suggesting that these taxa play a similar role in the trophic web of the Po and Danube Rivers. Regarding betweenness, there were no significant differences between the two rivers (median_po_ = 0.571, median_danube_ = 0.083, *n* = 30, Mann-Whitney U = 339, *p* = 0.102). The analysis showed that phytoplankton is important in both rivers (Table 3, Fig 4b), which is an evident result and can be extended to eutrophic rivers. Considering the nBC values of the shared species, some were more important in the Po River food web (*Carassius auratus*, *Abramis brama*, *Rutilus rutilus*), and others were more important in the Danube River food web (Chironomidae, Detritus, *Perca fluviatilis*). This means, that these nodes have a unique position within the network, frequently being on the shortest path between many nodes. Finally, based on both indices in the Po River, the non-native *Carassius auratus* is most important, whereas phytoplankton has higher betweenness than degree, and the non-natives *Abramis brama* and *Rutilus rutilus* are more important considering nDC than for nBC (Fig 4c). In the Danube River, phytoplankton and detritus are most important for both indices, whereas Chironomidae has higher betweenness than degree, and *Gymnocephalus cernua* is more important considering nDC than for nBC (Fig 4d). Contrary to the Danube River, these results suggest that non-native species are important trophic players in the Po River, at the expense of native species, confirming that the Po River is facing an invasive species crisis [46–49]. These nodes have been pointed out by network analysis and should be further examined for their ecological importance in each system. Some of the differences might be real ecological differences between the two rivers (e.g., the importance of *Carassius auratus* is higher in the Po than in the Danube), but others might simply be from the fact that they are better mapped in the Po than in the Danube (e.g., *Abramis brama*). Using connectance webs, the interpretations are limited and should only be used to point out important groups to be investigated in the future or those that are missing information.

**Table 3:** Local network metrics (nDC = normalized degree centrality, nBC = normalized betweenness centrality) for the shared nodes for the Po and Danube River food webs.

	Po		Danube	
Shared nodes	nDC	nBC	nDC	nBC
*Abramis brama*	0.459	4.631	0.008	0.156
ASELLIDAE/Asellus sp.	0.083	0.186	0.004	0
*Barbus barbus*	0.179	0.761	0.004	0
*Bosmina longirostris*	0.009	0.005	0.008	3.080
CAENIS/Caenis sp.	0.092	0.154	0.004	0
*Carassius auratus*	0.651	13.593	0.008	0.079
Chironomidae	0.147	1.244	0.029	13.316
Culicidae	0.069	0.854	0.008	0.004
*Cyprinus carpio*	0.142	0.616	0.008	0.729
Detritus	0.110	0.188	0.050	9.198
Diptera	0.110	0.795	0.008	0
Dytiscidae	0.069	0.058	0.004	0
Gammaridae	0.165	1.942	0.013	0.086
*Gymnocephalus cernua*	0.115	0.644	0.038	3.284
Hydracarina	0.064	0.091	0.004	0
Hydrophilidae	0.110	0.058	0.004	0
HYDROPSYCHIDAE/Hydropsyche sp.	0.115	0.096	0.017	2.296
*Lepomis gibbosus*	0.284	1.453	0.004	0
Leptoceridae	0.110	0.276	0.004	0
*Moina micrura*	0.009	0.005	0.004	0
Ostracoda	0.151	2.503	0.013	0.073
*Perca fluviatilis_ADULTS/Perca fluviatilis*	0.087	0.615	0.038	7.286
Phytoplankton	0.193	11.669	0.159	25.632
POTAMANTHUS/Potamanthus luteus	0.087	0.120	0.008	0
*Rutilus rutilus*	0.454	4.534	0.025	1.408
*Sander lucioperca*	0.023	0.105	0.017	3.727
*Scardinius erythrophthalmus*	0.115	0.221	0.021	1.130
*Silurus glanis_LARGE/Silurus glanis*	0.028	1.030	0.008	0.729
Sphaeriidae	0.064	0.526	0.004	0
Unionidae	0.050	0.420	0.008	0.781

## Conclusions

In this study, we aimed to highlight the importance of integrating monitoring data with network analysis for an ecosystem-level approach: the connectance web of the Po River and its main three river sections was compiled. This approach marks the need for a replicated approach for rivers considering their entire courses. We also emphasize the importance of using comparative methodology in order to be able to draw similarities and point out differences between different ecosystems. The Danube River food web was compiled using the same methodology, but highlighted different groups based on network analysis. Using aggregation, each network can be used for different purposes, but we recommend using the most aggregated versions for future modelling work (e.g., quantified networks, energy fluxes between the main compartments). We also found that the Sankey graphs (purely for visualization) and the REGE structural analysis nicely complement each other.

## Supporting information

S1 Table List of references for fish, invertebrates and plankton data in the three Po River sections. (DOCX)

S2 Table Data (predator-prey list) for each river section, the master network, and the aggregation steps (T1-T5). Node names can be found in Table 1.
(XLSX)
