# Supplementary material for: Spatial variability of the Po River food web and its comparison with the Danube River food web
Source: PLoS One. 2023 Jul 14;18(7):e0288652. doi: 10.1371/journal.pone.0288652 (PMC10348563; doi:10.1371/journal.pone.0288652)
Supplement: S1 Table — (DOCX) [file pone.0288652.s001.docx]

| **Taxa** | **Lower Po River** | **Middle Po River** | **Upper Po River** |
| --- | --- | --- | --- |
| Phytoplankton | Tavernini, Silvia, Enrica Pierobon, and Pierluigi Viaroli, ‘Physical Factors and Dissolved Reactive Silica Affect Phytoplankton Community Structure and Dynamics in a Lowland Eutrophic River (Po River, Italy)’, Hydrobiologia, 669.1 (2011), 213–25 <https://doi.org/10.1007/s10750-011-0688-2 | | |
| Zooplankton | Mantovani S. (2006). La funzionalità ecologica in un ecosistema artificiale: la rete di canali di bonifica della Provincia di Ferrara. PhD thesis | Rossetti, Giampaolo, Francesca Tireni, Simona Viglioli, and Ireneo Ferrari, ‘Ricerche Ecologiche in Un Ambiente Acquatico Della Golena Del Po Nei Pressi Di Casalmaggiore’, 80.2003 (2004), 193–200 | Rossetti, Giampaolo, Marco Bartoli, Lucia Ariotti, and Pierluigi Viaroli, ‘Studio Idrobiologico Di Ambienti Acquatici Golenali Del Parco Fluviale Del Po e Dell ’ Orba ( Alessandria )’, 17.2 (2003), 53–64 |
| Macro-invertebrates | ARPAV, MONITORAGGIO DEGLI ELEMENTI DI QUALITA’ BIOLOGICA DEI CORSI  D’ACQUA DEL VENETO (2009). https://www.arpa.veneto.it/dati-ambientali/open-data/file-e-allegati/acque-interne/fiumi-eqb/macroinvertebrati | ARPAV, MONITORAGGIO DEGLI ELEMENTI DI QUALITA’ BIOLOGICA DEI CORSI  D’ACQUA DEL VENETO (2009). https://www.arpa.veneto.it/dati-ambientali/open-data/file-e-allegati/acque-interne/fiumi-eqb/macroinvertebrati | ARPA PIEMONTE, ATTIVITA’ ARPA NELLA GESTIONE DELLA RETE DI MONITORAGGIO DELLE ACQUE SUPERFICIALI (2009) |
| Fish | Autority of the Po River Basin, Monitoraggio dell'ittiofauna e redazione della Carta ittica del Fiume Po, 2009 | | |
